# Towards Ultra Low-Cost Myoactivated Prostheses

**DOI:** 10.1155/2018/9634184

**Published:** 2018-10-04

**Authors:** Neethu Sreenivasan, Diego Felipe Ulloa Gutierrez, Paolo Bifulco, Mario Cesarelli, Upul Gunawardana, Gaetano D. Gargiulo

**Affiliations:** ^1^School of Computing, Engineering and Mathematics, Western Sydney University, NSW, Australia; ^2^DIETI, “Federico II” The University of Naples, Naples, Italy; ^3^The MARCS Institute, Western Sydney University, NSW, Australia

## Abstract

In developing countries, due to the high cost involved, amputees have limited access to prosthetic limbs. This constitutes a barrier for this people to live a normal life. To break this barrier, we are developing ultra-low-cost closed-loop myoactivated prostheses that are easy to maintain manufacture and that do not require electrodes in contact with the skin to work effectively. In this paper, we present the implementation for a simple but functional hand prosthesis. Our simple design consists of a low-cost embedded microcontroller (Arduino), a wearable stretch sensor (adapted from electroresistive bands normally used for “insulation of gaskets” against EM fields), to detect residual muscle contraction as direct muscle volumetric shifts and a handful of common, not critical electronic components. The physical prosthesis is a 3D printed claw-style two-fingered hand (PLA plastic) directly geared to an inexpensive servomotor. To make our design easier to maintain, the gears and mechanical parts can be crafted from recovered materials. To implement a closed loop, the amount of closure of prosthesis is fed back to the user via a second stretch sensor directly connected to claw under the form of haptic feedback. Our concept design comprised of all the parts has an overall cost below AUD 30 and can be easily scaled up to more complicated devices suitable for other uses, i.e., multiple individual fingers and wrist rotation.

## 1. Introduction

Upper extremity amputation, due to accidents, infections/disease, burns, and trauma, creates great challenges for the daily living of amputees [1]. The advancement of multifunctional prosthetics in recent years paved new paths for the normal living of amputees; unfortunately, the cost associated with manufacturing and maintenance is sometimes simply prohibitive for people living in developing countries where the cost of the prosthesis could exceed the year salary [2]. Some of the current prosthetic limb designs available in the market having exact replication of the human hand features' make the design extremely complex requiring constant fine-tuning, complex assembly, and constant maintenance [3]. As an example, the commercial hands with multiple gripping fingers and superior functions are expensive with costs up to USD 50,000 [4]. Off-note, despite the huge costs and richness of functionalities, even in countries where amputees can afford them, many of the highly functional upper-limb prosthetic devices available in the market have not been encountering the favor of the end users, resulting in a high rate of device abandonment. This is probably due to technological factors relating to discomfort, i.e., extensive training, issues related to durability, and other failures [5].

It is well understood that people with disabilities or amputees have more healthcare needs than others. Unfortunately, amputees as well as others with disabilities in developing countries are further disadvantaged by their economic limitations that in turn make them unsuccessful in getting proper care when needed [6]. In other words, access to proper healthcare is the major challenge faced by amputees in developing countries [7]. Due to lack of public health facilities and funds, the expenses of high functional artificial limbs are not affordable by amputees in the rural world. Additionally, most of the artificial limbs are typically designed for developed lifestyle making them unsuitable for the rural environment [8]. About the major provision of prosthetics services in developing countries, there are several factors involved in the choice of an artificial limb. Along with design viability, replacement and maintenance, level of comfort, overall cost, and cultural and religious backgrounds are factors, which decide the wide acceptance and use of artificial assistive technology [9, 10]. These choices are not always fulfilled due to the lack of funds and trained professional assistance [2]. Because of the increasing rate of amputations in developing countries the demand for prosthetic limbs is growing. The reduction of costs which is associated with artificial supports and limbs is the only way to eliminate these barriers [11]. With this work, we focus our effort on reducing these barriers for upper limbs and the hand.

Upper-limb prostheses (ULP) are generally classified into two categories based on their functionality: passive prostheses and active prostheses [12]. Active prosthetic devices with external power are desired by most of the users because of their functional suitability and appealing appearance. Surface electromyography (SEMG) signal is the most commonly used signal for these kinds of prosthesis [13]. SEMG prostheses are controlled by muscle contractions either from the residual limb or accessory muscles of the amputated limb (i.e., pectoral muscles) that, due to the amputation, despite being fully functional will not get used and eventually may also waste away. Once the user learns how to control the prosthesis, myoelectric arms are able to open/close the hand as well as control other degrees of freedom such as artificial wrist rotation and individual finger articulation. Critical to the functions of prosthesis are the SEMG signals acquisition which requires skin contact electrodes, instrumentation amplifiers, and powerful processing units [14–16]. In most cases, accurate measurement depends on type and placement of electrodes on residual muscle. Moreover, the electrodes are always prone to noise contaminations, so proper skin preparation is a must to maximize the signal to noise ratio [17, 18]. In addition, SEMG might need onboard processing and extensive user training [16]. This is because the signal has a characteristic of “spikes” corresponding to motor-units' activation that, aside from being highly dependent from the position where this is recorded [15], it needs at least to be integrated or filtered to extract its envelope (or RMS) that can be used as control signal [19].

A simple way of control for SEMG prosthesis can be achieved using a wearable band sensor, known as electroresistive bands (ERBs). The ERBs are a type of wearable cord transducer composed of a conductive rubber band [20, 21] with a diameter of about 2 mm and length of up to 1 m, having a resistivity in the range of 140–160 Ω/cm. The concept of ERBs is on the principle that, as the length of cord changes, its resistance will also change somewhat proportionally [22]. Although a nonperfect linear change of resistance with stretch has been demonstrated [23] for this sensor, nevertheless, it can be employed to acquire Analogous of Surface Electromyography (ASEMG) signals, where a change in ERB resistance is relatively proportional to the variations in muscle tension produced by the voluntary flexion. The volume shifts detected by the ERBs are more tolerant to changes in sensor location than conventional SEMG. Furthermore, they do not require any skin preparation; hence they are easy to wear and use with respect to the electrodes.

A viable example of the use of ERB sensor as ASEMG detection sensor was mentioned in [19] for the ULPs. The results show that the ERB sensor offers a vibrant control similar to the conventional EMG envelope. Moreover, it was able to provide both on-off easily and proportional control for the prosthesis. Although the control strategy depicted was new, the structure of prosthesis, its extraction, and processing are quite complex and overall cost involved in the production is comparatively high. This does not serve as the solution for the issue of vast majority amputees in the rural areas.

In this paper, we are proposing the design and mechanism of a simple, low-cost alternative to the existing ULPs [19] that uses ERBs and can achieve some hand basic tasks. The ASEMG signals acquired from ERBs reduce the computational power requested for the embedded system; hence, this can be replaced with an inexpensive control platform like Arduino [23, 24].

In other words, the controller recognizes muscle tensions from the digitized ASEMG and actuates proportionally the motor in the artificial hand. An additional ERB is employed as prosthesis position sensor, placed across the hand structure to provide a sensorial feedback [25, 26], and delivered to the user using a vibrating buzzer, providing a closed-loop system that costs as little as AUD 30. In this paper, we present the full implementation of our concept design together with the tests (performed on one single healthy, nonamputated, and volunteer) and the full bill of material required to reproduce the design.

## 2. Methods and Materials: Low-Cost Stretch Sensor Prosthetic Arm

Our design is composed of the following four parts:

1. ASEMG front-end
2. Arduino Nano controller
3. Mechanical claw with servo
4. User feedback circuit

### 2.1. ASEMG Front-End

The ASEMG front-end detection circuit consists of contactless ERBs sensors for sensing volumetric muscle activities and a Quad op-amp circuitry for the signal processing. In our design, we provided support for up to three ERB sensors, of which one is used to close the loop with the user (feedback). For this concept design, we only use one ERB as a volumetric sensor; a second ERB could be used to scale up the design, i.e., add more degrees of freedom. The ERBs are contactless wearable sensors; hence, skin preparation is not required; ERBs can be actually worn on the top of the user's clothes. In our prototype, we simply stitched the ERB onto a piece taken from a standard elasticated elbow support for simplicity of use. However, this can be attached using pins to existing clothes or bandages. Contrary to electrodes, ERBs are not as sensitive to external electromagnetic disturbances; therefore, the system can work in almost any environment.

**Figure 1:**
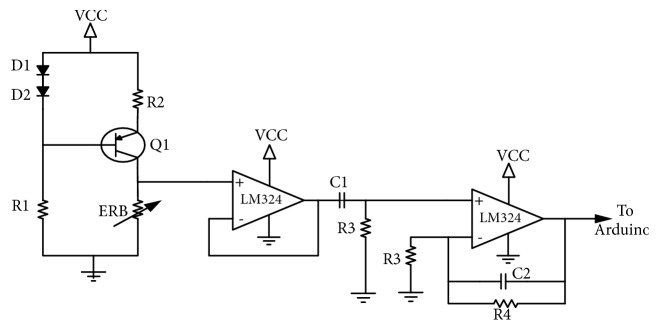
Circuit diagram of ERB sensors for ASEMG detection, limited to only one ERB (see text).

ERBs working principle is similar to the strain gauges. In other words resistance changes with the stretch, to enable this sensor to detect ASEMG as voltage changes that are directly readable by standard amplifiers; we employ a small DC polarization current. These feeble voltage changes are then amplified and fed to the ADC using a single operational amplifier. The full circuit for ERB sensors to detect SEMG is depicted in Figure 1.

In our design, a polarization DC current of ~1 mA (precision is not needed) is implemented using a simple biasing network composed by the resistors R1 and R2 and two small signal diodes as shown in Figure 1. The full part values are included in Table 1, bill of materials. As it is possible to see from Figure 1, the two diodes polarize a PNP transistor's base-emitter junction and set a constant current that depends on the diode's direct voltage value, the base-emitter voltage, and the emitter resistor; if the base resistance is adequately large to maintain the accurate polarization. The emitter current would be approximately equal to the current across the conductive ERB band, which is connected between the transistor's collector and return ground. The DC polarization circuit is designed around the BJT transistor BC557B, which is not critical and can be replaced by any of its equivalent.

The polarization current induces a voltage drop across the ERB, which is the function of the band's base resistance (DC mean) and from the stretching induced by the muscle contraction. This signal is conditioned, filtered, and amplified using two operational amplifiers from the four available inside the LM324 [25]. The LM324 is a general-purpose quadruple operational amplifier so one single chip is enough for our entire ASEMG circuitry; the LM324 is being selected due to its widespread availability and low-cost; moreover, because its characteristics are not critical, it can be easily replaced with any of the available general purposes operational amplifiers. As it is possible to see from Figure 1, one amplifier is just used as a voltage follower whose output is fed to a high-pass passive cell (measured corner frequency of ~0.2 Hz) and to this to an active low-pass cell with a measured gain of ~10 V/V and its measured corner frequency of ~235 Hz. The gain and cut-off frequencies of the signal in the conditioning circuits are not critical; so, in the prototype, we selected standard high-tolerance, low-cost passive components. Namely, the low-pass characteristic of the signal conditioning amplifier is required to guarantee antialiasing. With our component selection, we ensured a sample rate of ~500 Hz. As already mentioned, two identical circuits are used to polarize two ERBs that can be used as ASEMG sensors. When the user makes a targeted muscle flexion, the detected signals are converted via Arduino inbuilt ADC to drive the prosthesis.

### 2.2. Arduino Nano Controller

The Arduino Nano microcontroller is characterized by very low power consumption (Nanowatt technology) and comes with a user friendly veroboard and bread-board footprint. In our implementation, the Nano controller performs two-way communication (user to prosthesis and vice versa) with three basic activities: Analogue-to-Digital (A/D) conversion, Digital Signal Processing (DSP), and Digital-to-Analogue (D/A) conversion. The signals from ASEMG detection circuit are fed back to the mechanical hand after the processing within the controller. And, in return the microcontroller translates the reality existing in the prosthesis (position) and delivers a signal to the user in the form of haptic vibration.

The voltage outputs from ERBs are digitalized using the embedded 10-bit ADC inside the Arduino. Therefore, the output for muscle flexion is obtained as continuous digital counts, in which a threshold value is selected and set to cut off the weaker signals from spurious and unintentional muscle activities. The threshold level varies from person to person according to the capability of muscle flexion. The threshold to actuate the servomotor is drawn in a calibration phase where the user is asked to perform one voluntary reliable nonmaximal contraction; the value corresponding to the 50% of the measured ASEMG is then used as a threshold.

If the flexion count value is more than the set threshold, the controller calculates the time-based sampled average measured during the muscle contraction. This calculation produces a result similar to an RMS calculation; however, it is computationally lighter than a true RMS calculation across 50 samples. To identify the command of movement given by the user, a standard myoelectric pulse (threshold) is proposed, which is then in compared in terms of its RMS area with the myoelectric pulse detected to then compute the angle at which the servo motor would align. The threshold used in this work corresponds to a rectangular pulse with a duration of 500 ms and amplitude of the maximum value identifiable by the microcontroller's ADC, which in this case are 1023 counts for the Arduino Nano.

When the signal is above the threshold, a proportional PWM signal is generated for the actuation of the servomotor. If the signal is lower than the threshold value, the hand remains in the previous position. For prototyping purposes, a push button is provided for the user to change the direction of the claw movement. However, it would be easy to target a secondary muscle using the second provided ERB polarization/sensing circuitry to select the servomotor direction.

### 2.3. Mechanical Claw with Servo

**Figure 2:**
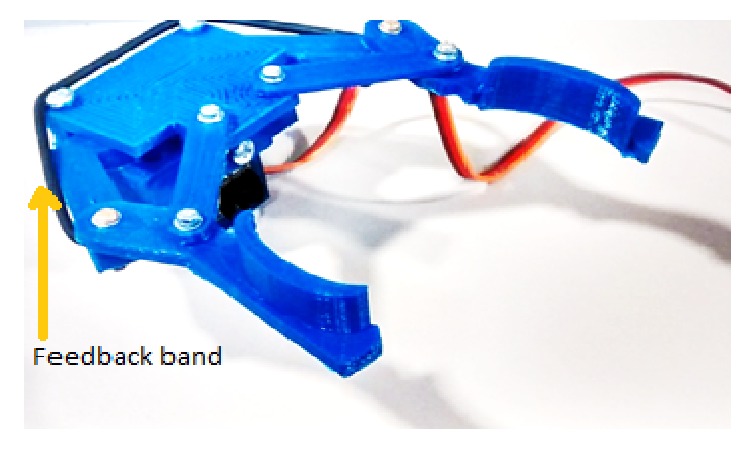
3D printed hand of the feedback ERB is visible (see text) and the connection to the circuitry has been removed to avoid clutter in the figure.

The processed signals from ASEMG unit through Arduino controller are used to drive the mechanical hand. The mechanical hand is a 3D printed claw-shaped structure with two fingers and a servo motor as shown in Figure 2. For the low-cost design of prosthesis, 3D printed hands are the best solution. The important advantage of 3D printed the design is that it can be improved with customized hand designs [26]. So we modified an open source project design available online (see download link in conclusion section), and all pieces of the prosthesis were in-house printed using Maker Bot 3D printer with PLA. The final product weighs 48 g and is very easy to assemble onto the servomotor.

A small DC servomotor MG992 is used for actuating the artificial hand. This motor is been selected for its low-cost, its high-torque, and a wide range of motion, of about 120 degrees (60 in each direction). In our design, despite the motor can rotate approximately 0 to 120 degrees due to the restrictions of hand design the maximum span obtained from the motor is limited to 45 degree. Once again, it is easy to replace this motor with an equivalent one. The total weight of the mechanical hand is approximately 103 g (including the servomotor).

**Figure 3:**
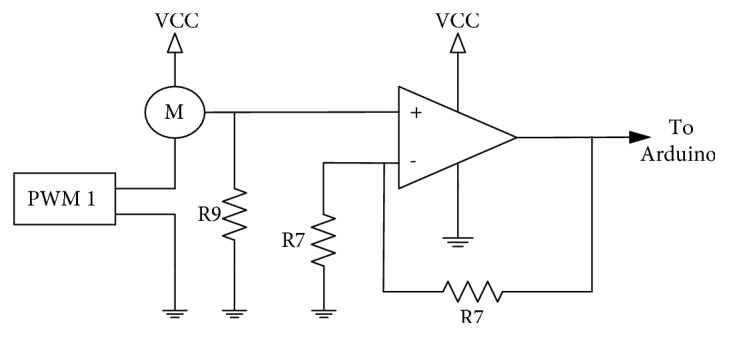
Circuit diagram of servomotor current sensing.

To detect an eventual motor stall and/or any overcurrent that could damage the prosthesis the current drawn by the servomotor is monitored using a sampling resistor and an amplifier (see Figure 3). Although this circuit is implemented onto the PCB, for this specific design we excluded this signal because we found that the sensory feedback control implemented is working well and we never experience the motor stalling issue during our experiments.

### 2.4. User Feedback Circuit

**Figure 4:**
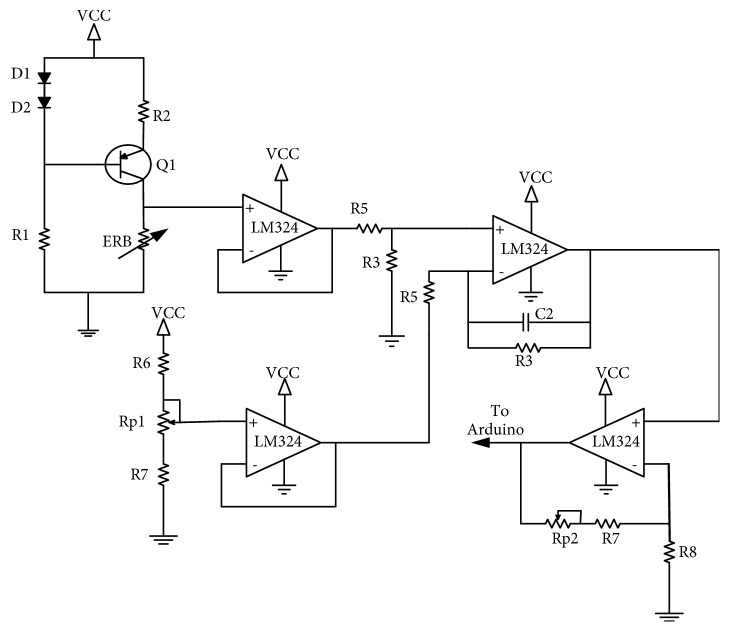
Circuit diagram of user feedback with span control.

To avoid unintentional injuries or damage to the prosthesis while gripping an object, we provided a real-time sensory feedback of the prosthesis aperture to the user [27, 28]. The sensory feedback to the user allows the control of the pressure applied when grasping an object. The degree of the prosthesis aperture is fed back to the controller using a third ERB. Contrary to the ERBs used for ASEMG, this circuit requires an additional signal conditioning stage (see Figure 4). As it is possible to observe in Figure 2, the ERB is placed across the two arms of the claw in such a way that it stretches when the prosthesis closes, the ERB, it is anchored in position by simple knots that are also used to make the necessary electrical connection to the ERB. The signal from this third ERB is used to activate a little buzzer placed on the user's body and its vibration amount gives information about the current aperture of the claw. To remove any ambiguity, a little amount of intermittent vibration is given when the claw is fully open. Full vibration is instead used to flag that the claw is fully closed.

**Figure 5:**
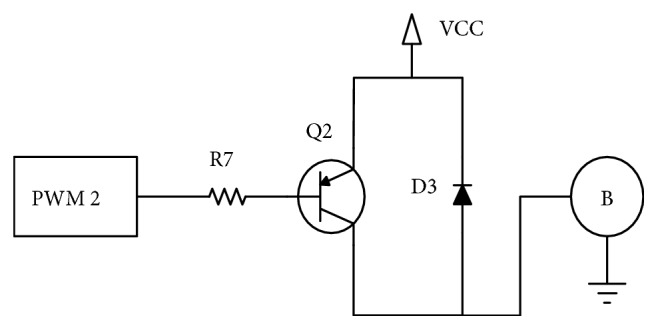
Circuit diagram of vibration buzzer in feedback control.

**Figure 6:**
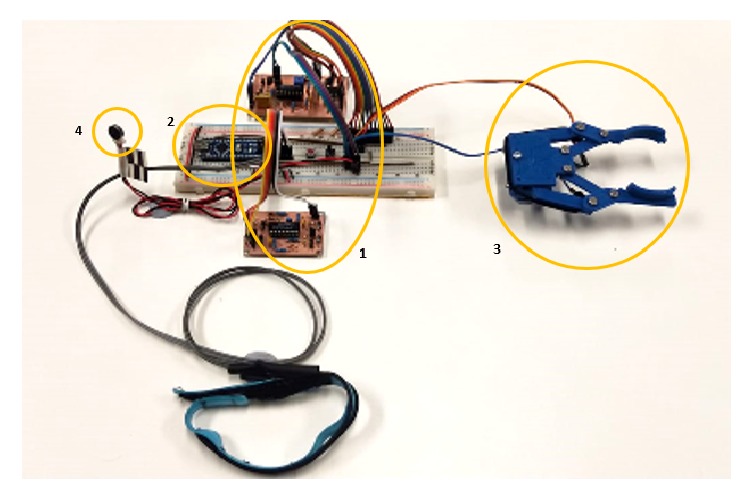
Device test setup (1: ASEMG circuit, 2: Arduino Nano, 3: mechanical claw with feedback band, and 4: vibration motor).

To customize the feedback according to the user preferences, additional controls over the zero span circuitry as in Figures 4 and 5 are used. Zero span circuitry allows the user to decide the level of full vibration and low vibration by means of gain adjustment. Adjusting the value of the potentiometer labeled Rp1, the user can change the vibration intensity level indicating the fully closed position to a comfortable value. The little vibration buzzer is directly driven by the Arduino using a simple BJT circuit as in Figure 5. The potentiometer Rp2 can be used to vary the gain of the signal conditioning circuit to adapt to different configurations, namely, the size/shape of the prosthesis. All circuit is made up of low-cost active components and materials and is powered directly from the 5 V DC supply output of the Arduino.  Figure 6 shows device test setup.

## 3. Results

**Figure 7:**
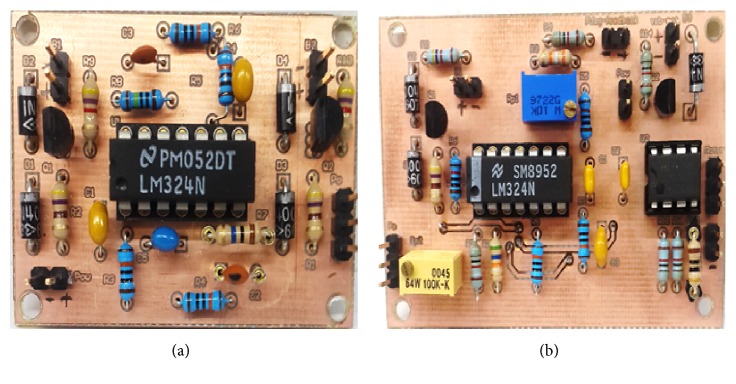
PCB assemblies: (**a**) ASEMG detection circuit; (**b**) user feedback circuit.

To proof feasibility of our low-cost ULP, we assembled the circuits on two small PCBs. The ASEMG detection PCB assembled is depicted in Figure 7(a). The PCB measures 33.5 × 47.5mm (ASEMG) and 44 × 65 mm (user feedback).  Figure 7(b) depicted the user feedback PCB fully assembled. Contrary to the ASEMG PCB, this circuit uses three copper bridges that for convenience we etched on the otherwise empty top copper layer. For the convenience of prototyping, for both PCBs, we used the top copper layer for the components' designators. This allowed us to use a milling machine and not a chemical etching process. The circuit was completely realized by low-cost readily available materials.

Although the full cost of the prototype is not prohibitive, because of the targeted population, maintenance can be simplified placing all the active components (transistors and integrated circuits) on sockets for prompt replacement. Moreover, a basic functional test for the mechanical part as well as for the ERBs will be included in the final version. The functional test for the motor will be fully implemented via software adding a simple hidden pushbutton to operate full claw closure hence ensuring that the motor is working correctly. To enable functional test of the ERBs front-end, a simple dual throw switch can be used to connect small potentiometer instead of the ERB under test and by varying the potentiometer wiper the operator can simulate ASEMG. Lastly, connecting the USB cable to the Arduino, raw data and processing results can be observed via the serial monitor.

**Figure 8:**
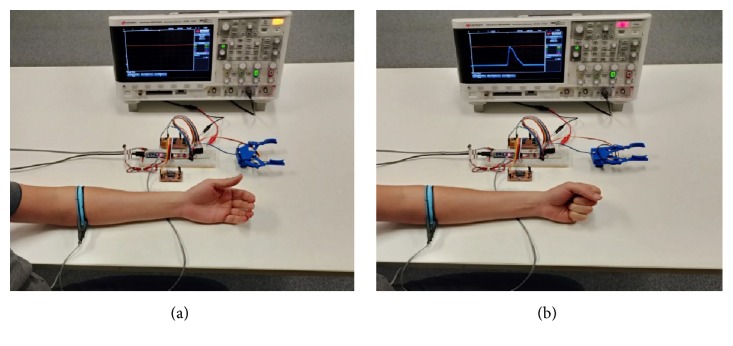
Prototype work mode: (**a**) hand fully open; (**b**) hand closing.

For this project we have not addressed circuit miniaturization and size optimization. However, these circuits could be stacked together with the Arduino and a small battery pack (i.e., 4 x AA) and strapped to the user elbow if present or hidden inside the cosmetic forearm for hand and wrist amputees. The full bill of materials, as mentioned, is reported in Table 1. The total cost is calculated with the current market value of components available in Australia. None of the components are critical so that each of them can be replaced by their similar characteristic items available in developing countries. The capture of the prototype working is provided in the Figure 8. As it is possible to infer form Figure 8, the prosthesis is been trialed on an able body healthy volunteer that has full control of the forearm muscles (target muscle). While this may seem to be a limitation, because of the embedded calibration procedure allowing the user to set and adjust the ASEMG detection threshold, muscle toning-up from the prosthesis use or change of target muscles is not problems. Our test user was able after few minutes of self-adjustments to switch target muscles from the forearm to the biceps.

**Figure 9:**
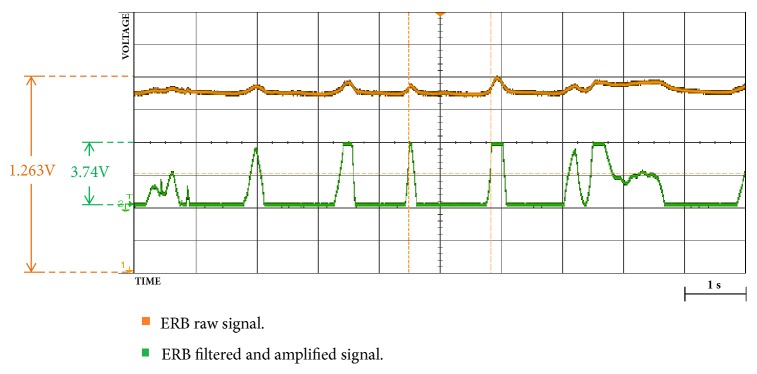
Raw signals of ERB sensor (yellow) and filtered and amplified signal (green) on continuous muscular variations.

**Figure 10:**
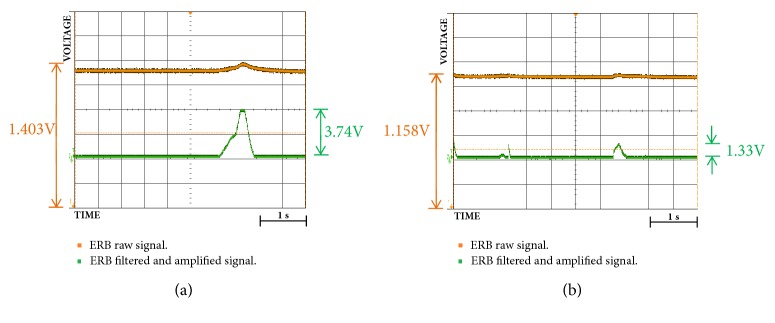
Oscilloscope results for signals of ERB sensor (yellow) and filtered and amplified signal (green): (**a**) signal after sudden flexion; (**b**) signals when fingers are moved.

**Figure 11:**
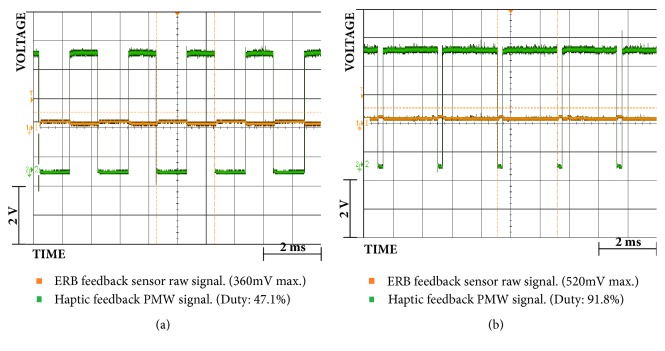
Raw signals of ERB feedback sensor (yellow) and PWM2 duty signals (green): (**a**) fingers full closed; (**b**) fully open.

**Figure 12:**
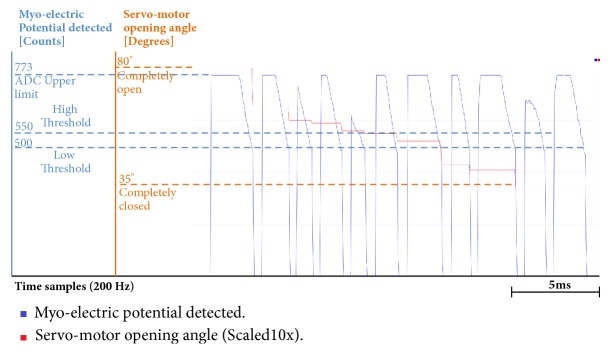
Arduino serial plotter capture for hand closing: red color represents standard myoelectric potential; blue color represents myoelectric potential detected by ERB bands.

**Figure 13:**
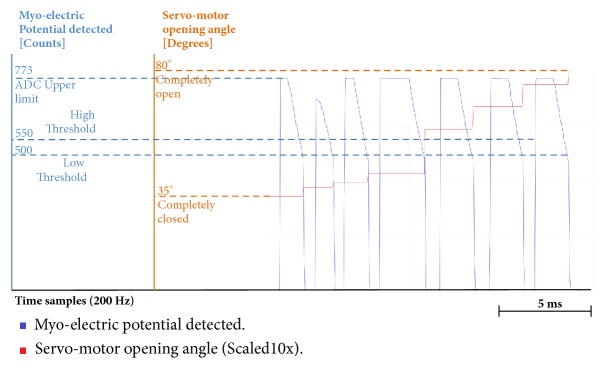
Arduino serial plotter capture for ERB band hand opening: red color represents standard myoelectric potential; blue color represents myoelectric potential detected by ERB bands.

**Figure 14:**
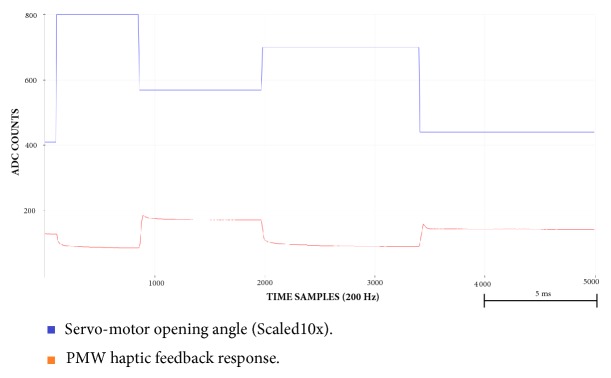
Arduino serial plotter capture for haptic feedback versus claw angle.

Also, through Figures 9–14, we report some of the signals recorded during our experiments with ASEMG and Feedback circuits. The results are produced by placing the ERB band on one of the author's normal hand during the voluntary flexion of upper wrist muscles to produce claws movements.  Figure 9 shows the raw volumetric shifts (top trace, yellow color) and its filtered counterpart during continue muscle activation. In Figure 10, the same signals were recorded during sudden strong flexion (panel (a)) and fingers movements (panel (b)). It is possible to observe from all the figures, the sensor is quite sensitive, hence, able to pick up even the smallest of the muscular volumetric shifts, i.e., from residual muscles on the stump.  Figure 11 shows an example of translation of the detected signals to PWM. The results obtained from the serial plotter of the Nano controller are depicted in Figures 12–14. Figures 12 and 13 represent the ERB band results (detected myopotentials) for opening and closing of the mechanical claw, whereas Figure 14 represents the haptic feedback obtained from the ERB band mounted on the claw.

The total current consumption of the prosthesis varies (see Table 2) and it is 76 mA when powered by the 5V outlet on the Arduino during the opening of the claw. The largest current consumption during opening is due to the mechanical drags inside the servomotor gears and the additional drag associated with the claw itself. Although this value seems quite high, these are measured during operation; in reality, the ULP consumes less power in average since that there will idle times.

## 4. Conclusions

Our main objective is to demonstrate that a low-cost and simple myoactivated prosthesis is attainable. In this brief paper we presented an implementation of such prosthesis that can be realized with a total cost of AUD 30 (see Table 1) with minimum components and compact assembly. The main advantage of our concept is that the prosthesis does not require contact with the skin to function properly; as additional benefit none of the components like BJTs and Op amps are strictly specific to the design, so they can be replaced with any of their equivalents. The control is simple and customizable with access to a computer and with minimal training (Arduino training). Despite the use of convenient 3D printing technology, the physical parts of the claw are so simple that they could be easily replaced with recovered materials (i.e., plywood).

Although a complete design could make a vast impact on the lives of deprived society [4], the functions demonstrated for the proposed design are limited to one degree of freedom and it is been tested only on a single able body volunteer. However, as already clarified, because the ASEMG sensor does not require complicate calculations and the ERBs are contactless and insensitive to target muscle changes or muscle strengthening up, this concept can be easily expanded. Moreover, we restricted the purpose of this prosthesis design to simple tasks like hold/grasp an object so that the hand model is in claw-like shape. Once again, the current focus of this paper is only on the mechanism and the functionality of the ERB band prosthesis circuitry and was tested only with the able-bodied person. That is a major limitation of the current study and needs to be addressed in future iterations with user-centered aspects.

Lastly, we would like to underline that the use of ERB as sensors in prosthesis could be discussed in a broader context where conventional electrodes play a major role and have been the reason for frustration and malfunction. This inexpensive sensor being contactless could be worn on the top of clothes and multiple sensors can be used to increase the prosthesis degree of freedoms. We conclude that this present concept design moves the myoactivated prosthesis towards a more affordable and easier to use reality. With all design compromises and trade-offs, the new model can provide a suitable solution towards the affordable prosthesis in developing countries.

In the appendix, we included the Arduino scripts for the threshold calibration. As well as the PCB Gerber files and the STL for the claw stile hand can be downloaded from https://github.com/neethurugma/Low-cost-prosthesis. These files together with the full bill of materials are everything that is required to reproduce the design.

## Figures and Tables

**Table 1:** Full bill of materials (prices are in Australian Dollars and correct to August 2018).

**Components**	**Type**	**Quantity**	**Amount**	**Total**
Diodes	D1, D2 - IN4148	4	0.05	0.2
	D3 - IN4001	1	0.08	0.08
Push button	MCDTS6	1	0.2	0.2
Resistors (Fixed)	R1 - 5K	3	0.08	0.24
	R2 - 470 Ω	3	0.05	0.15
	R3 - 100K	6	0.08	0.48
	R4 - 10M	2	0.2	0.4
	R5 - 10K	2	0.04	0.08
	R6 - 39K	1	0.08	0.08
	R7 - 1K	5	0.025	0.125
	R8 - 56K	1	0.024	0.024
	R9 - 10 Ω	1	0.05	0.05
Resistors(Variable)	Rp1 - 10K	1	1.19	1.19
	Rp2 - 100K	1	1.37	1.37
Capacitors	C1 - 7.9*μ*F	2	0.06	0.12
	C2 - 68pF	2	0.03	0.06
	C3 - 0.33*μ*F	1	0.08	0.08
BJT	Q1 - BC557B	2	0.3	0.6
	Q2 - BC548	1	0.05	0.05
Op Amps	LM324N	2	0.62	1.24
	LM358AP	1	0.4	0.4
Servo motor	MG996R	1	5	5
Vibrator (buzzer)r	Coin type	1	2	2
ERB bands	10cm	3	0.13/cm	3.9
3 D printed hand	PLA make(g)	48gm	0.08/gm	3.84
Arduino Nano	Atmega	1	6.95	6.95
				**Total = 29.04**

**Table 2:** Current consumption.

**Status**	**Current consumption**
Opening	76 mA
Closing	62 mA

## Appendix

### A.

This program reads analogue inputs from the ASEMG circuits as well as from the feedback circuit and produces the proportional servo/buzzer responses. The analogue values are also transmitted via serial port for debug/maintenance purposes.

#include <Servo.h>

Servo MyServo;

//Variable Declaration PINs

  int AInput7 = 7; //Analog Input pin A7 Band Position Control
  int AInput5 = 5; //Analog Input pin A5 Sesorial position Feedback
  int PWMOutput = 11; //PWM Output pin 11 Sesorial feedback
  int ServOut = 10; //Servo signal connected to pin D10
  int DirPIN = 3; //Digital Pin to change direction
  int CalPIN = 4; //Digital Pin to start the calibration protocol
  int ledPIN = 13; //Digital Pin with a led

//Global Variables

/*∗∗*Position Control*∗∗*/

  int AValueCtrl = 0;
  float RMS = 0;
  int ServAng = 0;
  int DeltaAng=0; //add/dim servo's angle
  int counter=0; //use to calculate Signal's RMS value

/*∗∗*Sensorial Feedback*∗∗*/

  int PWMDuty = 0; //Duty to control the vibrating motor
  int AValueFbk = 0; //analogue value read from the analogue channel
  int NSample=1; //counts to calculate the average
  float AnaAverg=0; //to calculate the average of the last 10 samples
  volatile int dir=-1; //to choose direction negative: close; positive: open;

void setup() {

//Activate serial transmision

Serial.begin(115200);

//set analog referece for the ADC

analogReference(EXTERNAL);

//Pin Setup

pinMode(A5, INPUT); //Used for the control signal

pinMode(A7, INPUT);//Used for the feedback signal(from the sensorial feedback sensor)

pinMode(ServOut, OUTPUT);//Claw psoition control

//Servo Setup

MyServo.attach(ServOut);

MyServo.write(80); //Initial position, Widely Open

//Interrupts Setup

attachInterrupt(digitalPinToInterrupt(DirPIN),Button Red,FALLING);

}//End Setup

void loop(){

/*∗∗∗∗∗∗*Position Control*∗∗∗∗∗∗*/

//Read a Value from the sensor:

AValueCtrl = analogRead(AInput7);

RMS = 0;

if (AValueCtrl>=550){

for (counter=0; counter<=100; counter++)

{ RMS = RMS + AValueCtrl;
  delay(5);
  AValueCtrl = analogRead(AInput7);
  if (AValueCtrl<500) {
  break;
};
};

RMS = RMS*∗*(counter+1)*∗*(0.001);

DeltaAng = 0.01*∗*RMS;

ServAng = ServAng + (DeltaAng*∗*dir);

if(ServAng>80){

ServAng = 80;

}; //Can't open wider!

if(ServAng<35){

ServAng = 35;

}; //Can't close narrower!

MyServo.write(ServAng);

}

/*∗∗∗∗∗∗*End Position Control*∗∗∗∗∗∗*/

/*∗∗∗∗∗∗*Sensory Feedback Control*∗∗∗∗∗∗*/

//Digital Low-pass Filter:

//Reads 10 values from the AInput, and take the average:

//Sampling rate, 5 ms

AnaAverg = 0;

for(int NSample=1; NSample<=20; NSample++){

//Read a Value from the sensor:

AValueFbk = analogRead(AInput5);

AnaAverg = AnaAverg + AValueFbk;

delay(5);

};

AnaAverg = AnaAverg/20;

/*∗∗*End Digital Low-pass Filter *∗∗∗∗∗∗∗∗*/

// Escale value:

PWMDuty = 0.25*∗*AnaAverg + 25;

if (PWMDuty<60) PWMDuty=70; //Min value that can be felt

if (PWMDuty>255) PWMDuty=255;//Max value in the motor

//Send the PMW value to the motor:

analogWrite(PWMOutput, PWMDuty);

/*∗∗∗∗∗∗*End Sensory Feedback Control*∗∗∗∗∗∗*/

}//End Loop

//Interruption Routine Service - Red Button

//When the button is pressed, the direction of the claw changes from opening to closing

//and viceverse

void ButtonRed(){

dir*∗*=-1;

if(dir<0){

Serial.println(“closing");

digitalWrite(ledPIN, HIGH);

}

else{

Serial.println(“opening");

digitalWrite(ledPIN, LOW);

};

};//End Button Red

### B.

This program shows the test part of the prosthetic controller.

#include <Servo.h>

Servo MyServo;

//Variable Declaration PINs

  int AInput7 = 7; //Analog Input pin A7 Band Position Control
  int AInput5 = 5; //Analog Input pin A5 Sesorial position Feedback
  int PWMOutput = 11; //PWM Output pin 11 Sesorial feedback
  int ServOut = 10; //Servo signal connected to pin D10
  int DirPIN = 3; //Digital Pin to change direction
  int CalPIN = 4; //Digital Pin to start the calibration protocol
  int ledPIN = 13; //Digital Pin with a led

//Global Variables

/*∗∗*Position Control*∗∗*/

  int AValueCtrl = 0;
  float RMS = 0;
  int ServAng = 0;
  int DeltaAng=0; //add/dim servo's angle
  int counter=0; //use to calculate Signal's RMS value

/*∗∗*Sensorial Feedback*∗∗*/

  int PWMDuty = 0; //Duty to control the vibrating motor
  int AValueFbk = 0; //analogue value read from the analogue channel
  int NSample=1; //counts to calculate the average
  float AnaAverg=0; //to calculate the average of the last 10 samples
  volatile int dir=-1; //to choose direction negative: close; positive: open;

/*∗∗*Code Timing*∗∗*/

  int TimeIni = 0;
  int TimeEnd = 0;
  int DeltaT = 0;
  bool Active = false;

void setup() {

// put your setup code here, to run once:

//Activate serial transmision

Serial.begin(115200);

//set analog referece for the ADC

analogReference(EXTERNAL);

//Pin Setup

pinMode(A5, INPUT); //Used for the control signal

pinMode(A7, INPUT);//Used for the feedback signal(from the sensorial feedback sensor)

pinMode(ServOut, OUTPUT);//Claw psoition control

//Servo Setup

MyServo.attach(ServOut);

MyServo.write(80); //Initial position, Widely Open

//Interrupts Setup

attachInterrupt(digitalPinToInterrupt(DirPIN),Button Red,FALLING);

attachInterrupt(digitalPinToInterrupt(CalPIN),Button Black,FALLING);

}//End Setup

void loop() {

/*∗∗∗∗∗∗*Position Control*∗∗∗∗∗∗*/

//Read a Value from the sensor:

AValueCtrl = analogRead(AInput7);

//Serial.print(“AValueCtrl: ");

//Serial.print(AValueCtrl);

//Serial.print(“, ");

//Serial.println(10*∗*ServAng);

RMS = 0;

if (AValueCtrl>=550){

//TimeIni = millis(); to calculate the time

for (counter=0; counter<=100; counter++)

{ RMS = RMS + AValueCtrl;
  // Serial.print(RMS);Serial.print(“, ");
  //Serial.println(AValueCtrl);
  // Serial.println(counter);
  delay(5);
  AValueCtrl = analogRead(AInput7);
  //Serial.print(AValueCtrl);
  //Serial.print(“, ");
  //Serial.println(10*∗*ServAng);
  if (AValueCtrl<500) {
  break;
}; };

//TimeEnd = millis();

//DeltaT= TimeEnd-TimeIni; end calculating the time spent

// Serial.print(“Delta T = ");Serial.println(DeltaT);

RMS = RMS*∗*(counter+1)*∗*(0.001);

//Serial.print(“RMS = ");Serial.print(RMS); Serial.print(“/");Serial.println(counter);

DeltaAng = 0.01*∗*RMS;

//Serial.print(“DeltaAng= ");Serial.println(DeltaAng);

ServAng = ServAng + (DeltaAng*∗*dir);

if(ServAng>80){

ServAng = 80;

}; //Can't open wider!

if(ServAng<35){

ServAng = 35;

}; //Can't close narrower!

MyServo.write(ServAng);

//Serial.print(“ServAng= ");

//Serial.print(AValueCtrl);

//Serial.print(“, ");

//Serial.println(10*∗*ServAng);

}

/*∗∗∗∗∗∗*End Position Control*∗∗∗∗∗∗*/

/*∗∗∗∗∗∗*Sensory Feedback Control*∗∗∗∗∗∗*/

//Digital Low-pass Filter:

//Reads 10 values from the AInput, and take the average:

//Sampling rate, 5 ms

AnaAverg = 0;

for(int NSample=1; NSample<=20; NSample++){

//Read a Value from the sensor:

AValueFbk = analogRead(AInput5);

AnaAverg = AnaAverg + AValueFbk;

delay(5);

};

AnaAverg = AnaAverg/20;

/*∗∗*End Digital Low-pass Filter *∗∗∗∗∗∗∗∗*/

// Escale value:

PWMDuty = 0.25*∗*AnaAverg + 25;

if (PWMDuty<60) PWMDuty=70; //Min value that can be felt

if (PWMDuty>255) PWMDuty=255;//Max value in the motor

//Send the PMW value to the motor:

analogWrite(PWMOutput, PWMDuty);

// Print value via serial

//Serial.print(“Position= ");Serial.print(AnaAverg);

Serial.print(10*∗*ServAng);

Serial.print(“, ");

//Serial.print(“Duty= ");

Serial.println(PWMDuty);

/*∗∗∗∗∗∗*End Sensory Feedback Control*∗∗∗∗∗∗*/

}//End Loop

//Interruption Routine Service - Red Button

//When the button is pressed, the direction of the claw changes from opening to closing

//and viceverse

void ButtonRed(){

dir*∗*=-1;

if(dir<0){

Serial.println(“closing");

digitalWrite(ledPIN, HIGH);

}

else{

Serial.println(“opening");

digitalWrite(ledPIN, LOW);

};

};//End Button Red

//Interruption Routine Service - Black Button

//When the button is pressed, the configuration mode for the feedback starts

void ButtonBlack(){

int Value = 0;

//Blink three times, to start calibration

digitalWrite(ledPIN, LOW);

delay(500);

digitalWrite(ledPIN, HIGH); //1

delay(300);

digitalWrite(ledPIN, LOW);

delay(300);

digitalWrite(ledPIN, HIGH); //2

delay(300);

digitalWrite(ledPIN, LOW);

delay(300);

digitalWrite(ledPIN, HIGH); //3

delay(300);

digitalWrite(ledPIN, LOW);

/*∗∗∗∗∗∗∗∗∗∗∗∗∗∗∗∗∗∗∗∗∗∗∗∗∗∗∗∗∗∗∗∗∗∗∗∗∗∗*/

do{

//Read the analogue port and change resistance in Zero Pot until it reaches 200 counts

Value = analogRead(AInput5);

delay(100);

}while(Value!=200);

//Blink once to continue calibration

digitalWrite(ledPIN, HIGH); //1

delay(300);

digitalWrite(ledPIN, LOW);

delay(300);

/*∗∗∗∗∗∗∗∗∗∗∗∗∗∗∗∗∗∗∗∗∗∗∗∗∗∗∗∗∗∗∗∗∗∗*/

do{

//Read the analogue port and change resistance in Span Pot until it reaches 950 counts

Value = analogRead(AInput5);

delay(100);

}while(Value!=950);

//Blink Twice to finish calibration

digitalWrite(ledPIN, HIGH); //1

delay(300);

digitalWrite(ledPIN, LOW);

delay(300);

digitalWrite(ledPIN, HIGH); //2

delay(300);

digitalWrite(ledPIN, LOW);

delay(300);

/*∗∗∗∗∗∗∗∗∗∗∗∗∗∗∗∗∗∗∗∗∗∗∗∗∗∗∗∗∗∗∗∗∗∗*/

loop();//Starts main program again

};//End Black Red
